# Amyloid Imaging in Aging and Dementia: Testing the Amyloid Hypothesis *In Vivo*

**DOI:** 10.3233/BEN-2009-0232

**Published:** 2009-10-21

**Authors:** G. D. Rabinovici, W. J. Jagust

**Affiliations:** ^1^Memory and Aging CenterUniversity of California San FranciscoSan FranciscoCAUSA; ^2^Department of NeurologyUniversity of California San FranciscoSan FranciscoCAUSA; ^3^Helen Wills Neuroscience InstituteUniversity of California BerkeleyBerkeleyCAUSA; ^4^Lawrence Berkeley National LaboratoryBerkeleyCAUSA

**Keywords:** Amyloid imaging, PET, PIB, beta-amyloid, brain aging, MCI, Alzheimer’s disease

## Abstract

Amyloid imaging represents a major advance in neuroscience, enabling the detection and quantification of pathologic protein aggregations in the brain. In this review we survey current amyloid imaging techniques, focusing on positron emission tomography (PET) with ^{11}carbon-labelled Pittsburgh Compound-B (^11^C-PIB), the most extensively studied and best validated tracer. PIB binds specifically to fibrillar beta-amyloid (A*β*) deposits, and is a sensitive marker for Aβ pathology in cognitively normal older individuals and patients with mild cognitive impairment (MCI) and Alzheimer’s disease (AD). PIB-PET provides us with a powerful tool to examine in vivo the relationship between amyloid deposition, clinical symptoms, and structural and functional brain changes in the continuum between normal aging and AD. Amyloid imaging studies support a model in which amyloid deposition is an early event on the path to dementia, beginning insidiously in cognitively normal individuals, and accompanied by subtle cognitive decline and functional and structural brain changes suggestive of incipient AD. As patients progress to dementia, clinical decline and neurodegeneration accelerate and proceed independently of amyloid accumulation. In the future, amyloid imaging is likely to supplement clinical evaluation in selecting patients for anti-amyloid therapies, while MRI and FDG-PET may be more appropriate markers of clinical progression.

## 1. Introduction

The development of techniques for imaging brain amyloid protein aggregations *in vivo* represents a major breakthrough in clinical neuroscience. Amyloid imaging provides our first "real time" glance into the evolution of brain pathology with aging and disease, and allows us to ask questions about the relationship between amyloid pathology and clinical measures in ways that were never before feasible. The advent of amyloid imaging comes at an opportune time for Alzheimer's disease (AD) research, as the "amyloid hypothesis" is increasingly challenged due to the failure of therapies targeting beta-amyloid (A$\beta$) to demonstrate efficacy in clinical trials [1]. Amyloid imaging allows us to critically readdress questions about the relationship between A$\beta$ aggregation and AD that have not been definitively answered by post-mortem studies, including the significance of A$\beta$ aggregation in cognitively normal individuals, and the relationship between the distribution and burden of amyloid pathology and clinical features of AD [2–6]. Furthermore, amyloid imaging has the potential to detect A$\beta$ pathology in very mildly affected or even asymptomatic individuals, when amyloid-modifying therapies may have the highest likelihood of success [7]. In this review we will survey the currently available amyloid imaging markers, focusing on the most extensively validated tracer, $^{11}$C-labelled [$N$-methyl]-2-(4'-methylaminophenyl)-6-hydroxybenzothiazole (Pittsburgh Compound-B, PIB). We will then review the rapid-

ISSN 0953-4180/09/$17.00 © 2009 – IOS Press and the authors. All rights reserved ly expanding literature applying PIB-PET to study cognitively normal volunteers and patients with mild cognitive impairment (MCI) and AD, and summarize what PIB-PET studies have taught us thus far about the relationship between amyloid, aging and dementia.

## 2. Amyloid imaging markers

The first published attempt to image amyloid *in vivo* was reported by Friedland and colleagues, who administered a technetium-99 labeled anti-A$\beta$ monoclonal antibody fragment to AD patients imaged with single photon emission computed tomography (SPECT) [8]. While this attempt was not successful, as the antibody proved too large to effectively cross the blood-brain barrier, it instructed future attempts to develop amyloid imaging agents, focusing efforts on small molecules with increased brain permeability.

The first successful amyloid imaging agent employed in humans was $^{18}$fluoro-labelled1,1-dicyano-2-[6-(dimethylamino)-2-naphthalenyl]propene (FDDNP), a fluorinated derivative of a nonspecific cell membrane dye [9]. FDDNP binds *in vitro* to amyloid conformations of A$\beta$, tau and prion protein [9,10], and in 2002 Shoghi-Jadid and coworkers demonstrated increased tracer binding on positron emission tomography (PET) in 9 patients with AD compared to 7 matched controls [11]. Tracer retention was highest (30% greater than the pons reference region) in medial temporal cortex, hippocampus and amygdala, regions which typically show dense neurofibrillary tangles (NFTs), and was also increased 10–15% above baseline in frontal, temporal and parietal cortex, regions which typically show both A$\beta$ plaques and NFTs. In one patient who later came to autopsy, increased FDDNP-PET signal during life co-localized to regions with significant plaque and tangle pathology post-mortem [12].

The most widely studied amyloid imaging agent is PIB, an analog of the amyloid-binding dye Thioflavin-T. *In vitro*, PIB binds specifically to extracellular and intravascular fibrillar A$\beta$ deposits in post-mortem AD brains [13–16]. At PET tracer concentrations, PIB does not appreciably bind to other protein aggregates such as NFTs or Lewy bodies [13,16,17]. PIB does bind non-specifically to white matter, likely due to delayed clearance of the lipophilic compound from white matter [18]. In 2004 Klunk and colleagues reported the first human study of PIB-PET [19]. Thirteen of 16 mild AD patients demonstrated elevated PIB-PET signal in frontal, parietal and lateral temporal cortex and the striatum, compared to a reference region in the cerebellum (which shows low levels of fibrillar A$\beta$ pathology even in advanced AD [20]). One of nine cognitively normal controls also showed elevated cortical binding [19]. At a group level, PIB cortical signal in patients was 1.5–2 times higher in patients compared to controls [19]. PIB has subsequently been shown to correlate inversely with cerebrospinal fluid (CSF) levels of A$\beta_{1-42}$, another *in vivo* marker of A$\beta$ pathology [21–23]. Most convincingly, detailed post-mortem study of two patients who showed elevated PIB-PET signal during life demonstrated strong correlations between regional *in vivo* PET signal and *in vitro* measures of A$\beta$ pathology (both plaques and vascular amyloid) found at autopsy [16,24].

FDDNP and PIB have different binding properties and distinct advantages and disadvantages. FDDNP binds to both A$\beta$ and tau amyloid conformations, and thus accounts for the full spectrum of AD pathology. The highest region of FDDNP signal in AD is typically the medial temporal cortex, a region with high NFT but low plaque burden [12]. As expected, PIB signal in medial temporal cortex is not elevated in AD patients compared to controls [19]. FDDNP also binds to amyloid conformations of prion protein [10], and thus may have *in vivo* utility in prion diseases [25]. On the other hand, PIB appears to be highly specific for A$\beta$ amyloid, which makes it a better tracer for diagnostically discriminating between AD and non-A$\beta$ dementias [26,27]. FDDNP binding can be disrupted by commonly used non-steroidal anti-inflammatory drugs [28], while similar drug interactions have not yet been reported for PIB. From a practical point of view, FDDNP is labeled with $^{18}$fluourine which has a relatively long half-life of 110 minutes, allowing for potential commercial production and distribution, as is done for $^{18}$F-fluorodeoxyglucose (FDG). PIB is labeled with carbon-11, which has a half-life of 20 minutes, limiting its use to PET centers with cyclotrons. However, the short half-life is a potential advantage for research, since it allows PIB imaging to be easily combined on the same day with other PET tracers (e.g. FDG).

In a study directly comparing FDDNP and PIB-PET in the same subjects (14 AD, 11 MCI and 13 controls), Tolboom and coworkers reported a nearly 10-fold higher binding potential in AD for PIB compared to FDDNP [29]. Global cortical PIB discriminated the three diagnostic groups, whereas global cortical FDDNP only differentiated between AD and controls. Across groups, PIB correlated more strongly with cognition than FDDNP. Specific (cortical) ver-

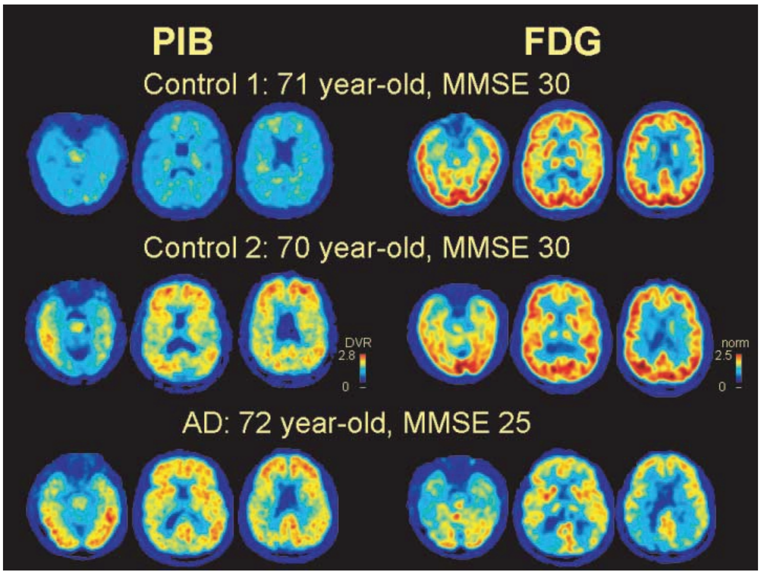

Fig. 1. PIB and FDG in controls and Alzheimer's disease (AD). Control 1 demonstrates only non-specific PIB binding to white matter (top row, left), while Control 2 shows diffuse cortical and striatal PIB binding (middle row, left) that is indistinguishable from binding in a patient with AD (bottom row, left). However, only the AD patient demonstrates reduced glucose metabolism on FDG-PET (bottom row, right), whereas both controls show intact resting metabolism (top and middle row, right). PIB images are parametric distribution volume ratio (DVR) images using a cerebellar reference region, while FDG images are normalized to mean activity in pons. All images are in neurologic orientation. MMSE – Mini-Mental State Exam.

sus non-specific (white matter and brainstem) binding could be readily discerned by visual inspection of PIB images (for demonstration see Fig. 1 from our laboratory), whereas quantification was needed to distinguish subjects with elevated FDDNP. Shin and colleagues reported similar results in 10 AD patients and 10 controls studied with both tracers, though they found a smaller discrepancy in effect size in favor of PIB [30]. Both groups found elevated medial temporal binding with FDDNP but not with PIB, highlighting the complementary information gained by combining both tracers. A recent *in vitro* study compared FDDNP and PIB binding to sub-adjacent sections from AD pathologic specimens and found comparatively weak binding of FDDNP compared to PIB [31]. Of the two agents, only PIB has been quantitatively validated *in vivo* compared to autopsy [16,24]. Published reports of FDDNP have thus far originated in three centers and encompass a limited number of subjects [12,29,30], whereas PIB studies have been performed in an estimated 3,000 participants in over 40 centers around the globe [32].

A number of additional $^{11}$C-labeled PET tracers have been studied in humans as potential amyloid imaging markers [33,34]. Though preliminary results have been encouraging, it seems unlikely at this point that another $^{11}$C-labeled tracer will surpass $^{11}$C-PIB as the gold-standard amyloid PET tracer. However, the short half-life of $^{11}$C is prohibitive in terms of adopting $^{11}$C-PIB into clinical practice and limits its availability for research. Thus, most current efforts are focused on developing a fluourine-18 labeled tracer of comparable quality to $^{11}$C-PIB. Three potential agents currently being studied are $^{18}$F-AH110690 (a 3'-fluoro analog of PIB), the stilbene derivative $^{18}$F-BAY94-9172 (which performed comparably to $^{11}$C-PIB in a preliminary study in AD and controls [35]), and $^{18}$F-AV-45 [32,35,36]. Efforts also continue to develop amyloid imaging agents for SPECT and MRI (reviewed by Nordberg [37]). In October 2008 an advisory committee for the United States Food and Drug Administration recommended that autopsy-confirmed studies be required to validate amyloid imaging agents prior to their approval for clinical use (http://www.aan.com/elibrary/neurologytoday/?event=home.showArticle&id=ovid.com:/bib/ovftdb/00132985-200811200-00003).

## 3. Amyloid imaging in normal controls

For the remainder of this review we will focus on PIB, which is the best validated amyloid imaging marker, and survey what we have learned from PIB-PET studies about the natural history and impact of amy-

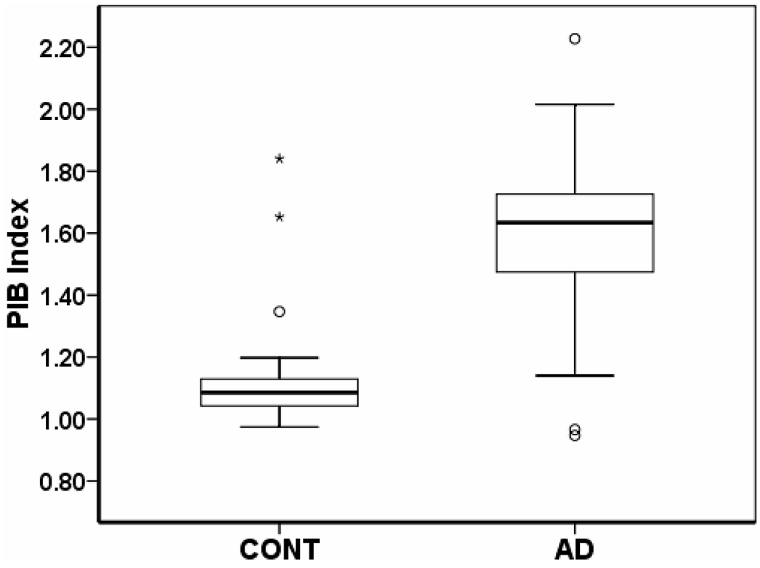

Fig. 2. PIB Index in controls and AD. Box and whisker plots represent median (line), $25^{th}$ and $75^{th}$ percentile (box) and 1.5 times interquartile range (IQR, whiskers) of PIB Index in controls recruited from the Berkeley Aging Cohort (CONT, N = 30, mean age 73.7 ± 6.7, MMSE 29.2 ± 1.0), and AD patients evaluated at the University of California San Francisco (N = 38, age 68.4 ± 10.6, MMSE 22.8 ± 4.5). Outliers (PIB Index between 1.5 and 3 IQR) are denoted by a circle and extreme outliers (PIB Index greater than 3 IQR) by an asterisk. PIB Index represents mean distribution volume ratio (DVR) in frontal, parietal, lateral temporal and cingulate cortex. The control with the second highest PIB Index is featured in Fig. 1, middle row.

loid deposition, beginning with cognitively normal older individuals and culminating in dementia. Autopsy studies in the past decade have demonstrated that significant A$\beta$ amyloid can be found post-mortem in 25%–45% of cognitively normal older individuals, and that the extent and distribution of pathology may be indistinguishable from that found in AD [4,5,38]. The first PIB-PET study in humans suggested that PIB may be sensitive to this pathology, with one of nine normal controls showing elevated tracer binding [19]. Subsequent studies have consistently detected elevated tracer binding in a subset of normal older volunteers, with the proportion of "PIB-positive" cases ranging from 10%–30% depending on the age of the cohort and the threshold for defining PIB-positivity [19,39–45]. In contrast, elevated binding has not been reported in young normal controls [39]. Binding in carriers of the apolipoprotein E4 (Apo E4) genotype is higher than in non-carriers, with suggestion of a gene dose effect [44]. In most studies, PIB uptake in older controls falls along a continuum between the very low binding seen in young controls and the high levels seen in AD (Fig. 2) [39–45]. The pattern of binding is similar to AD and diffusely involves prefrontal cortex, lateral and medial parietal regions, lateral temporal cortex and striatum. Binding patterns can be more focal, and a number of studies suggest preferential uptake in prefrontal cortex and posterior cingulate/precuneus [27,39,42,45], similar to the regions of earliest amyloid deposition noted in autopsy studies [46]. A number of controls show focal binding in occipital cortex, a pattern suggestive of vascular amyloid deposition [27]. Finally, a minority of older controls show a distribution and burden of PIB binding that is essentially indistinguishable from that seen in AD (Figs 1, 2) [39,40,42,43]. The high rate of PIB-positivity in normal controls underscores that a positive PIB scan cannot be interpreted without a careful clinical evaluation, and emphasizes that amyloid imaging alone must not serve as a surrogate for a clinical diagnosis of AD or dementia.

A major unresolved issue in AD research is whether cognitively normal persons with amyloid deposition are on a trajectory towards AD, or whether the pathology is "benign" in these individuals. Cross-sectional post-mortem studies have yielded equivocal results in this regard, with some studies finding no association between post-mortem amyloid pathology and antemortem cognitive performance, and other studies reporting subtle cognitive deficits in amyloid-positive subjects [4, 38,47,48]. PIB-PET has several potential advantages over autopsy-based studies in tackling this important question. First, amyloid imaging allows for more rigorous quantification of the distribution and burden of amyloid, and enables direct comparison of these measures to simultaneously-derived cognitive metrics, in contrast to the significant delay between behavioral assessment and autopsy often seen in post-mortem studies. Furthermore, PIB-PET has the potential for higher throughput than autopsy studies, and, critically, allows investigators to follow amyloid-positive controls longitudinally to determine their cognitive trajectory.

Cross-sectional studies evaluating the relationship between PIB binding and cognition in older control subjects have yielded seemingly conflicting results. When subjects are dichotomized into "PIB-positive" and "PIB-negative" groups, most studies have not found significant differences in cognitive performance [27,39,41,42], with the exception of a single study that found lower episodic memory scores in the PIB-positive group [40]. However, the sample sizes in these studies were relatively small (range 20–45 subjects, of whom 4–9 were classified as PIB-positive), such that they may have been under-powered to find a subtle effect. In contrast, most studies that evaluated PIB as a continuous variable have found significant negative correlations between PIB uptake and episodic memory scores [40,41,43,45], though these relationships may rely on outliers and appear to vary between cohorts [43]. These results are not necessarily contradictory, and suggest that continuous regressions may be more sensitive to the relationship between PIB and cognition, at least until larger samples are studied and more objective (i.e. pathology-based) cut-offs for PIB-positivity are determined.

Two studies have reported negative correlations in normal controls between PIB uptake and hippocampal volumes measured by MRI [41,43]. When PIB binding, hippocampal volume and episodic memory scores were included in the same model using either multilinear [43] or logistic regression [41], hippocampal atrophy appeared to mediate the relationship between PIB binding and memory loss. Other studies have found reductions in whole-brain volume [49] and cortical thickness in AD-specific regions [50] in PIB-positive normal controls compared to their PIB-negative counterparts. Studies are currently underway to investigate the relationships between PIB binding in controls and functional imaging markers of AD, such as glucose hypometabolism and decreased resting state connectivity [51,52].

While prospective longitudinal data on PIB-positive controls are not yet available, Villemagne and colleagues retrospectively examined cognitive test scores over 6–10 years in 34 subjects enrolled in the Melbourne Healthy Aging Study [45]. They found that PIB-positivity was more common in subjects with declining cognitive test scores (70%) than in those with stable scores (17%). In contrast, the PIB-positive control in the first PIB-PET study has remained cognitively stable over five years [53]. Overall, the preponderance of currently available data suggest that at a group level, PIB uptake in nondemented subjects is associated with decreased episodic memory performance, and with structural and functional brain changes suggestive of incipient AD. Several large longitudinal studies are currently underway to test this hypothesis more definitively.

## 4. Amyloid imaging in mild cognitive impairment

Amyloid imaging can potentially identify patients with MCI who already show A$\beta$ aggregation and are thus in the early clinical phase of AD, and separate them from patients with alternative substrates for cognitive impairment [54]. Such separation may have prognostic implications, assuming that patients with underlying AD are at higher risk of progressing to dementia. Dividing MCI patients into more biologically homogeneous groups may also facilitate their inclusion in clinical trials for AD-specific therapies, allowing these treatments to be tested in patients earlier in the disease course, when therapies may have a higher likelihood of success [7].

At a group level, MCI patients show PIB uptake that is intermediate between AD patients and controls [22,27,40,41,43,55–57]. However, PIB binding levels in MCI in most studies are bimodal, with a majority of patients demonstrating AD-like uptake levels, a minority showing low-control level binding, and a small number of patients falling in the intermediate range. At an individual subject level, 52%–87% of MCI patients show elevated PIB binding, depending on the criteria used to diagnose MCI and the threshold used to define PIB-positivity [22,27,40,41,43,55–57]. Patients meeting criteria for amnestic MCI are more likely to be PIB-positive than patients with non-amnestic MCI [22,40], and PIB-positivity is also more common in Apo E4 carriers [40]. Two studies suggest that PIB is more sensitive than CSF A$\beta_{1-42}$ levels in detecting amyloid pathology in MCI [22,57], though this may be an artifact of choosing more sensitive cut-offs for PIB than for CSF A$\beta$.

Cross-sectional comparisons of PIB-positive and PIB-negative MCI patients reveal lower performance on episodic memory tasks in PIB-positive patients in some studies [40] but not others [27,41]. As in controls, when PIB is examined as a continuous variable, negative correlations are found between tracer binding and episodic memory scores [22,40,43], and between PIB uptake and hippocampal volume [41,43]. In terms of prognosis, pooled results from three studies with longitudinal data (range: eight months to two years) reveal that 17/17 MCI patients that progressed clinically or converted to dementia were PIB-positive [22, 40,57]. While more longitudinal studies are needed, current data suggest that amyloid imaging may provide important prognostic information in MCI, and will help select MCI patients that are candidates for AD-specific therapies. These sentiments are reflected in the proposed new AD research criteria, in which the diagnosis of AD can be made in the absence of dementia in patients with an amnestic syndrome and PET evidence of amyloid deposition [58].

## 5. Amyloid imaging in Alzheimer's disease

The initial "proof of concept" studies of amyloid imaging with PIB-PET focused on detecting A$\beta$ amyloidosis in patients clinically diagnosed with AD. As expected, the vast majority of AD patients show elevated PIB retention as judged either by visual assessment or by quantification of tracer uptake (Figs 1, 2) [19,26, 27,41,59,60]. Using clinical diagnosis as a gold standard, the sensitivity of PIB-PET for AD has been reported as 80%–100%, with most studies reporting sensitivities of 90% or greater [19,26,39,60]. The significance of a "negative" PIB scan in a patient clinically diagnosed with AD is not yet clear due to the lack of post-mortem data. The proportion of PIB-negative scans in AD is very similar to the fraction of patients clinically diagnosed with AD at dementia referral centers who are subsequently found to have an alternative pathology at autopsy [61], suggesting that many PIB-negative scans in AD may represent "true" negatives. One pathology-confirmed "false-negative" PIB result has been reported thus far, involving a patient with A$\beta$ plaques on frontal brain biopsy who showed low PIB binding when studied with PET twenty months later [62]. While the histopathologic characterization of this case was limited, it seems likely that PIB will fail to detect amyloid deposits below a certain threshold, and that, rarely, PIB may fail to bind to certain human amyloid confirmations, as has been reported in transgenic AD mice [63]. Therefore, while preliminary studies based on clinical diagnosis are encouraging, the precise sensitivity and specificity of PIB-PET for AD pathology need to be determined by post-mortem studies.

The distribution of PIB uptake in patients with AD closely mirrors the distribution of A$\beta$ fibrillar plaques found at autopsy [64,65]. Using either voxel-wise or region-of-interest analysis, highest tracer uptake is consistently found in prefrontal cortex, precuneus and posterior cingulate cortex, followed closely by lateral parietal and temporal cortex and striatum, with relatively lower tracer uptake in occipital cortex, globus pallidus and thalamus (Fig. 1) [19,27,41,60,66]. Primary sensorimotor and visual cortex are relatively spared, as are medial temporal cortex, hippocampus and amygdala. Cerebellar gray matter shows very little tracer uptake, mirroring post-mortem findings [20] and making the cerebellum a convenient reference region for normalizing PET counts across subjects [55,67]. One study reported slightly higher cortical binding in ApoE4 carriers versus non-carriers [68]. In most patients the distribution of tracer uptake is diffuse and symmetric, with very high inter-correlation between regions [69].

Interestingly, patients with familial AD due to presenilin-1 (PS1) mutations and familial AD and cerebral amyloid angiopathy (CAA) due to reduplication of the amyloid precursor protein (APP) show an atypical pattern of PIB retention, with very high tracer uptake in the striatum and relatively low cortical uptake [70–72]. This distinct binding pattern contrasts with the more extensive cortical A$\beta$ pathology found at autopsy in patients from the same pedigrees [70,71]. The explanation for this unusual binding pattern is not clear. Striatal binding is found in asymptomatic PS-1 mutation carriers years before expected symptom onset, suggesting that striatal amyloid deposition may be an early feature of familial AD [70]. However, this explanation does not account for striatal-predominant binding in patients with moderate dementia in the same pedigrees. An alternative explanation is that PIB has a lower binding affinity for A$\beta$ plaques associated with specific AD mutations, perhaps due to microstructural differences in amyloid, as has been described in AD transgenic mice (including APP/PS1 mice) [63]. These conformational differences may interfere to a greater degree with PIB binding to the compact plaques in neocortex than to the relatively diffuse plaques in striatum, leading to an artifact of greater tracer binding in striatum. Again, autopsy studies are needed to distinguish between these possibilities.

Most studies have found either weak or absent correlations between PIB uptake and clinical markers of AD severity such as episodic memory scores, Mini-

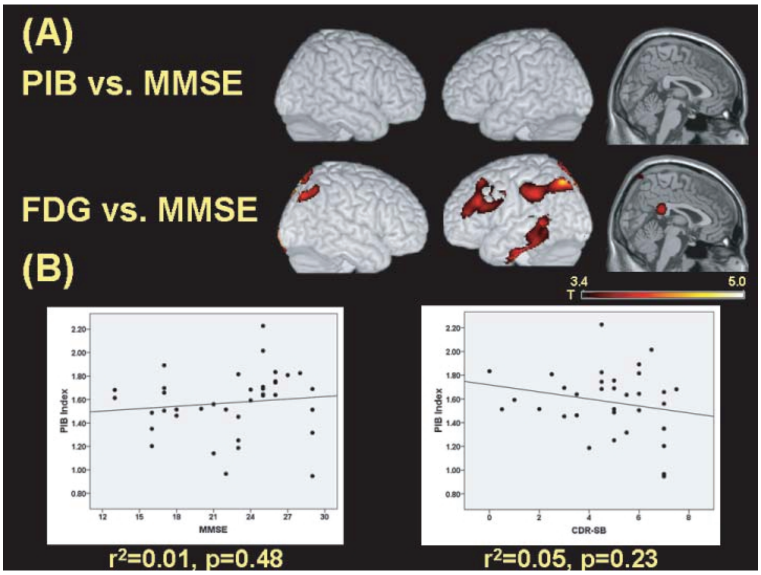

Fig. 3. Relationships between PIB, FDG and AD severity. (A) Voxel-wise parametric regressions between PIB and MMSE (top row) and FDG and MMSE (bottom row) in 38 AD patients (age 68.4 ± 10.6, MMSE 22.8 ± 4.5), controlling for age, sex and education. Images are thresholded at $p < 0.001$, uncorrected for multiple comparisons. MMSE correlated positively with FDG in bilateral parietal and left frontal, lateral temporal and posterior cingulate cortex, while no significant correlations (positive or negative) were found between PIB and MMSE at this threshold. (B) Regression of PIB Index (mean distribution volume ratio (DVR) in frontal, parietal, lateral temporal and cingulate cortex) versus MMSE (left panel) and Clinical Dementia Rating Sum of Boxes (CDR-SB, group mean 4.7 ± 2.1, right panel) reveal no significant correlations between amyloid burden and measures of AD severity.

Mental State Exam (MMSE) [73] and Clinical Dementia Rating [74] (Fig. 3) [19,27,40,60,75–77]. Studies that found correlations often pooled controls and AD patients together, or included PIB-negative AD subjects (that tend to have high cognitive scores and low levels of functional impairment) in the analysis [40,41, 60,75]. Correlations significantly weakened or disappeared when controls and PIB-negative AD subjects were excluded from the analysis [40,60]. The cross-sectional association between PIB and cognitive measures is weaker in AD than in MCI and normal controls [40], and grows weaker yet with AD progression [75]. Across studies, clinical measures are more tightly linked to brain atrophy (measured by MRI) and glucose hypometabolism (FDG-PET) than to PIB uptake (Fig. 3) [41,60,75,77,78]. These findings are consistent with the majority of clinicopathological studies that have not found a strong correlation between A$\beta$ amyloid plaque burden and AD severity [2,3,79].

The regional relationship between PIB uptake and brain structural and functional changes in AD is complex [19,22,41,60,75]. High PIB binding is associated with hypometabolism and atrophy in lateral and medial (posterior cingulate/precuneus) parietal regions and lateral temporal cortex, but not in frontal cortex or striatum. Conversely, hypometabolism and atrophy in the absence of PIB binding are seen in medial temporal cortex and hippocampus. Primary sensorimotor and visual cortices are spared of amyloid and also remain metabolically and structurally intact. The biological mechanisms that underlie these relationships are not well understood. The metabolic and structural sparing of striatum despite a high amyloid burden may be explained by the relatively diffuse (and thus less neurotoxic) nature of striatal A$\beta$ deposits [80]. However, this rationale cannot explain the relative sparing of prefrontal cortex, where neuritic A$\beta$ pathology can be extensive [65,81]. Putative explanations for the markedly selective temporoparietal patterns of atrophy and hypometabolism seen in AD in the face of diffuse A$\beta$ pathology include: greater synaptic reserve in prefrontal cortex [82,83], relative vulnerability of posterior brain regions due to differences in lifetime metabolic activity or gene expression patterns [84–86], and differences in the distribution of pathology not captured by PIB, such as soluble A$\beta$ and NFTs [46,87]. Hippocampal and medial temporal injury in the setting of low A$\beta$ deposition may be largely driven by NFT pathology [88], although disconnection from medial parietal cortex and high local levels of soluble A$\beta$ have also been postulated as potential mechanisms to explain this discrepancy [85, 87].

PIB-PET shows low (~5%) test-retest variability, and is thus suitable to measure longitudinal changes in amyloid burden [67,75]. All 16 patients with AD included in the original PIB-PET study underwent repeat imaging 2.0 ± 0.5 years later [75]. At a group level, there were no significant increases in global or regional PIB binding, though there was considerable variability between subjects, with many showing small increases in PIB and, paradoxically, a number showing decreases in uptake. There was a clear dissociation in individual patients between amyloid load, which remained relatively stable, and clinical status, which declined considerably in a number of patients. The clinical decline was much better reflected by decreasing glucose metabolism measured by FDG-PET. The rate of cognitive decline correlated with changes in FDG but not in PIB. Not surprisingly, correlations between PIB and cognition and PIB and FDG that were observed in this cohort at baseline were no longer significant at follow-up. Jack and colleagues found similar results when examining changes in PIB binding and brain atrophy over one year in 21 normal controls, 32 patients with MCI, and 8 patients with AD [78]. At a group level there was a small but significant increase in PIB over one year. The rate of increase was similar across diagnostic groups and across brain regions. In contrast, brain atrophy rates (as measured by changes in ventricular volume) were highest in AD, intermediate in MCI and lowest in controls. Longitudinal clinical decline correlated strongly with brain atrophy rates but did not correlate with changes in PIB.

## 6. Amyloid imaging in other dementia syndromes

PIB can detect A$\beta$ deposition in other dementia syndromes associated (to varying degrees) with beta-amyloidosis, including CAA, dementia with Lewy bodies (DLB) and posterior cortical atrophy (PCA) [27, 89–93]. PIB can also be used to exclude atypical presentations of AD in clinical syndromes typically associated with non-A$\beta$ pathology such as frontotemporal lobar degeneration (FTLD), primary progressive aphasia (PPA) and prion disease [25–27,94,95,97]. Patients with CAA show relatively higher occipital PIB retention than AD patients [89,98], consistent with the typical anatomic distribution of this pathology at autopsy [99]. Patients with DLB show a deposition pattern similar to AD, though overall amyloid levels tend to be lower and inter-subject variability is higher [27,91,92]. Amyloid aggregation in DLB may be associated with greater cognitive deficits [91,100] and more rapid disease progression [27]. Single case studies have reported asymmetric left hemisphere PIB uptake in PPA [90] and posterior-predominant PIB uptake in PCA [90,93]. However, at a group level we have found PIB uptake in PPA and PCA to be diffuse, symmetric and indistinguishable from the pattern seen in AD, whereas atrophy and glucose hypometabolism patterns are asymmetric and more closely match the clinical phenotype [94, 101]. Non-amnestic presentations of AD are relatively common at dementia referral centers [102], and PIB may have a future role in selecting patients with atypical clinical symptoms who may be candidates for anti-A$\beta$ therapies [103].

## 7. PIB-PET and the amyloid hypothesis: Integrating the data

Taken together, PIB-PET studies support a model in which A$\beta$ aggregation is an early event on the path to dementia. Amyloid accumulation begins insidiously and progresses slowly in a subset of older individuals. While the cognitive effects of A$\beta$ deposition in these healthy older people is debatable, structural and functional imaging studies suggest that the protein has a deleterious effect that appears similar to AD in several respects. By the time patients reach the MCI phase amyloid accumulation already approximates the levels seen in AD, a process which may take over two decades [78]. As patients progress to mild dementia, clinical decline and neurodegeneration (as reflected by MRI and FDG-PET) accelerate and proceed in tight relation to each other, but apparently independent of amyloid accumulation, which has either reached a plateau or continues to progress very slowly.

The data used to derive this model have inherent limitations. First, more rigorous work is needed to establish the quantitative relationship between PIB binding and A$\beta$ pathology at various disease stages. It is conceivable, for example, that PIB binding may reach a ceiling at high plaque density, and thus fail to capture the progression of pathology beyond a certain disease stage. Second, most data are derived from cross-sectional studies, and need to be verified by longitudinal investigations. Finally, PIB binds only to fibrillar A$\beta$, and apparent dissociations between PIB uptake and other disease measures may be accounted for by soluble forms of A$\beta$ that are not reflected by PIB-PET. With these limitations in mind, this working model of amyloid aggregation suggests that the optimal time to initiate therapies targeting A$\beta$ (at least in its fibrillar form) may be in the pre-MCI stage, when cognition is still intact, and neurodegeneration is mild and appears linked to amyloid levels. By the time patients are in the mild stages of AD or perhaps even in the MCI stage, other pathologic processes that are independent of fibrillar A$\beta$ may already be in motion, and the therapeutic window for anti-plaque interventions may already be closed.

## 8. Conclusion

In the past five years amyloid imaging has established itself alongside MRI and FDG-PET as a core neuroimaging tool for the evaluation of brain aging and dementia. In particular, PET imaging with $^{11}$C-PIB appears to be a sensitive and specific diagnostic marker for underlying A$\beta$ amyloidosis, and can detect pathology in patients with early or atypical symptoms as well as in asymptomatic older adults. PIB-PET has provided us with our first *in vivo* glance at the dynamic relationship between amyloid deposition, clinical symptoms, and structural and functional changes in the brain in the continuum between normal aging and AD. A number of promising $^{18}$F-labelled imaging markers are currently under development, and these will allow broader application of amyloid imaging to clinical practice and research. In the future, A$\beta$ imaging will likely supplement clinical evaluation in selecting patients for anti-amyloid therapies both during drug development and in the clinic, while alternative imaging techniques such as MRI and FDG-PET may be more appropriate markers of clinical progression. The development of *in vivo* biomarkers for other critical elements of AD pathogenesis such as soluble A$\beta$, tau, acetylcholine and brain inflammation would further inform our understanding of the complexities of the disease in humans, and ultimately assist in developing and testing disease-modifying and perhaps preventive treatments for AD.

## Acknowledgments

GDR is supported by the National Institute on Aging (NIA) K23-AG031861, Alzheimer's Association NIRG-07-59422 and the John Douglas French Alzheimer's Foundation. The original research presented in this manuscript was supported by NIA grants AG027859, P01-AG1972403 and P50-AG023501 and by Alzheimer's Association ZEN 08-87090.
